# Population-specific molecular features and clinical significance of urinary tumor DNA mutations in Chinese patients with newly diagnosed non-muscle-invasive bladder cancer

**DOI:** 10.3389/fonc.2026.1914617

**Published:** 2026-10-07

**Authors:** Zugang Sun, Run Xie, Jian Pan, Yiping Zhu, Yijun Shen, Chengyuan Gu, Chunguang Ma, Shengming Jin, Huina Wang, Dingwei Ye, Yao Zhu, Junlong Wu

**Affiliations:** 1 Department of Urology, Guangyuan Central Hospital of Sichuan Province, Guangyuan, China; 2 Department of Urology, Fudan University Shanghai Cancer Center, Shanghai, China; 3 Department of Nursing, Fudan University Shanghai Cancer Center, Shanghai, China; 4 Shanghai Medical College, Fudan University, Shanghai, China; 5 Acornmed Biotechnology Co., Ltd, Beijing, China

**Keywords:** Chinese population, FGFR3 mutations, non-muscle-invasive bladder cancer, qPCR, TERT promoter mutations, urinary liquid biopsy

## Abstract

**Background:**

Non-muscle-invasive bladder cancer (NMIBC) is characterized by high recurrence rates, highlighting the need for effective, non-invasive diagnostic and risk stratification tools. Although urinary biomarkers are increasingly used worldwide, molecular data specific to Chinese NMIBC populations remain limited. This study explores the potential utility of TERT promoter and FGFR3 hotspot mutations detected in urinary tumor DNA (utDNA) in a prospective Chinese cohort.

**Methods:**

We prospectively enrolled 106 newly diagnosed NMIBC patients between February 2023 and August 2023 at Fudan University Shanghai Cancer Center (ChiCTR2300076543). First-morning urine samples were collected prior to TURBT. A PCR-based UI-Seek assay was employed to detect hotspot mutations in the promoter region (M1: c.-146 C>T, C250T; M2: c.-124 C>T, C228T) and FGFR3 gene (M1: c.742 C>T, p.R248C; M2: c.746 C>G, p.S249C).

**Results:**

The overall mutation rates for TERT promoter and FGFR3 were 49.06% and 15.09%, respectively, with a combined sensitivity of 55.66%. Compared with published study cohorts from Western countries and Japan, Chinese NMIBC patients showed differences in molecular mutation profiles. Notably, the TERT-M2 mutation was associated with tumor aggressiveness, with significant correlations observed for T1 stage (P = 0.011), high-grade tumors (P = 0.011), and high/very high EAU risk categories (P < 0.001). In the subgroup of patients with rapid recurrence within 12 months, TERT promoter mutations (particularly M2), high-grade histology, T1 stage, and high/very high EAU risk categories were significantly enriched. Conversely, FGFR3 mutations showed no significant correlation with clinicopathological parameters. Patients were stratified into early recurrence (≤12 months, n = 14) and long-term recurrence-free (>24 months, n = 40) groups. T1 stage, high tumor grade, high/very high-risk stratification, and TERT mutations (especially the TERT-M2 subtype) were significantly associated with early recurrence (P < 0.05), whereas sex, tumor multiplicity, presence of CIS, and FGFR3 mutations showed no significant correlation with recurrence timing (P > 0.05).

**Conclusion:**

This study characterizes the mutational landscape of urinary tumor DNA (utDNA) in Chinese patients with non-muscle invasive bladder cancer (NMIBC). Our findings suggest that utDNA-based detection of TERT promoter mutations, particularly the M2 subtype, and FGFR3 mutations may provide complementary molecular information for recurrence risk assessment. Larger prospective multicenter studies are warranted to validate these findings and evaluate the clinical potential of utDNA as a non-invasive molecular tool in NMIBC management.

## 1 Introduction

Bladder cancer is one of the most common malignant tumors of the urinary system worldwide. According to 2024 estimates from the Global Cancer Observatory (GLOBOCAN), there were approximately 614, 300 new cases and 220, 600 deaths attributed to bladder cancer globally. It ranks as the 9th most common malignancy in terms of incidence and the 13th in terms of mortality, underscoring its substantial global burden of morbidity and mortality (1).In China, the disease burden of bladder cancer is also non-negligible. Studies have shown that in 2019, there were approximately 100, 000 new cases of bladder cancer in China (82, 700 in males and 17, 300 in females) and 40, 000 deaths (35, 800 in males and 4, 300 in females), with an increasing trend in age-standardized incidence rates. Projections indicate that by 2030, the number of new cases will increase to approximately 150, 000 and deaths to 54, 000, suggesting a continuously growing disease burden (2).Urothelial carcinoma (UC) is the predominant pathological subtype of bladder cancer, among which approximately 75%–85% of patients are diagnosed as non-muscle-invasive bladder cancer (NMIBC) at initial presentation. Although patients with NMIBC have a relatively favorable prognosis after transurethral resection of bladder tumor (TURBT), they carry a high risk of recurrence (5-year recurrence rate: 50%-70%) and a certain risk of progression (10%–15% progressing to muscle-invasive bladder cancer), thus necessitating long-term intensive follow-up (3). This imposes a heavy burden on both patients and the healthcare system.

In recent years, with advances in genomic research on bladder cancer, many key genes and hotspot mutations related to the occurrence and development of bladder cancer have been identified, including TERT promoter and FGFR3 hotspot mutations. TERT (Telomerase Reverse Transcriptase) is the catalytic subunit of telomerase; two hotspot mutations (C228T and C250T) in its promoter region can create new ETS transcription factor binding sites, leading to TERT transcriptional activation and re-expression of telomerase, thereby endowing cancer cells with unlimited proliferative potential (4). Mutations in FGFR3 (Fibroblast Growth Factor Receptor 3) can induce constitutive activation of its tyrosine kinase domain, which further activates the downstream MAPK and PI3K/AKT signaling pathways, promoting cell proliferation and inhibiting apoptosis (5). In-depth research on these core driver genes not only lays a theoretical foundation for understanding the molecular pathogenesis of bladder cancer but also provides critical biomarker targets for exploring liquid biopsy based on urinary tumor DNA (utDNA). Notably, a large number of studies have shown that bladder cancer patients of different ethnic groups may have differences in genomic characteristics (such as key genes and mutation frequencies), and such differences also highlight the importance of fully considering the population genetic background in clinical translation (6, 7).

Currently, cystoscopy combined with urinary cytology is considered the “gold standard” for diagnosing bladder cancer. However, cystoscopy is an invasive procedure with poor patient compliance, while urinary cytology, although non-invasive, has very low sensitivity for detecting low-grade tumors (only 20%–40%), significantly limiting its use in early screening and recurrence monitoring (8). In recent years, urine-based liquid biopsy technology has made significant advances in the non-invasive diagnosis of bladder cancer, demonstrating considerable value in early detection, recurrence monitoring, and treatment efficacy evaluation. Among these methods, urinary tumor DNA (utDNA), which originates directly from the tumor microenvironment, has become a focal point of research in bladder cancer liquid biopsy (9). Multiple studies have confirmed the clinical utility of utDNA-based detection of key gene mutations for predicting non-muscle invasive bladder cancer (NMIBC) recurrence and for companion diagnostics in neoadjuvant therapy for muscle-invasive bladder cancer (MIBC), thereby supporting precision treatment decisions, including targeted immunotherapy regimens and bladder-preserving strategies (10). Nevertheless, current research on urine-based liquid biopsy for bladder cancer faces two major limitations: first, most studies are based on single-population cohorts and lack cross-ethnic comparative analyses, making it difficult to generalize their findings to the Chinese population; second, most detection models constructed using next-generation sequencing (NGS) technology involve dozens of genes, resulting in complex procedures, high costs, and challenges in interpretation, which hinder their widespread adoption in primary healthcare settings.

Based on the above context, the present study utilized a prospective clinical trial cohort of patients with non-muscle invasive bladder cancer (NMIBC) and employed the UI-Seek Urine Assay, which is based on PCR technology, to detect TERT promoter and FGFR3 hotspot mutations in urinary tumor DNA from Chinese patients newly diagnosed with NMIBC.This study assessed associations between these mutational features and clinicopathological parameters, compared our results with datasets from other published study cohorts, and explored their potential relevance among Chinese patients with NMIBC.

## 2 Patients and methods

### 2.1 Study population

A total of 106 patients with newly diagnosed NMIBC were enrolled from the Department of Urology at Fudan University Shanghai Cancer Center between February 2023 and August 2023. All patients participated in a prospective clinical trial (ChiCTR2300076543). Each case was histopathologically confirmed as urothelial carcinoma via cystoscopic biopsy, with tumor stages classified as Ta or T1, and no evidence of muscle invasion or distant metastasis.

Inclusion criteria: (1) Age ≥ 18 years; (2) Pathologically confirmed NMIBC, with no prior chemotherapy, radiotherapy, immunotherapy, or other anti-tumor treatments; (3) Voluntary participation with signed written informed consent; (4) Complete clinical data available.

Exclusion criteria: (1) History of other malignant tumors; (2) Severe organ dysfunction (hepatic or renal) or coagulation disorders; (3) Inadequate urine samples or failed DNA extraction; (4) Incomplete clinical data preventing further analysis.

This study was approved by the Ethics Committee of Fudan University Shanghai Cancer Center (Ethics No.: 2023-FS-KY-012) and registered with the Chinese Clinical Trial Registry (ChiCTR2300076543). All study procedures were conducted in strict accordance with the Declaration of Helsinki and relevant clinical research guidelines.

### 2.2 Pathological diagnosis

All tissue specimens obtained from transurethral resection of bladder tumor (TURBT) were fixed in 10% neutral buffered formalin, paraffin-embedded, sectioned, and stained with hematoxylin and eosin (H&E). All slides were independently reviewed and diagnosed by two senior pathologists from the Department of Pathology at Fudan University Shanghai Cancer Center. Tumor staging was determined according to the 8th edition of the TNM staging system by the American Joint Committee on Cancer (AJCC). Tumor grading was classified as low-grade or high-grade in accordance with the 2022 World Health Organization (WHO) Classification of Tumors of the Urinary and Male Genital Systems. Tumor size, multiplicity, presence of concomitant carcinoma *in situ* (CIS), and rare histological subtypes were systematically recorded. All patients were stratified into low-, intermediate-, high-, and very high-risk groups for recurrence and progression based on the 2022 European Association of Urology (EAU) guidelines.

### 2.3 Sample collection, processing, and DNA isolation

First-morning urine samples of 100–200 mL were collected from all patients prior to surgery and stored in disposable urine collection tubes containing a DNA preservative (Tianjin Xiangxin Medical Device Co., Ltd., Tianjin Medical Device Registration No. 20220525). All samples were transported to the laboratory within 24 hours at 4 °C. For sample processing, urine specimens were centrifuged at 1600 rpm for 20 minutes, and cell pellets were collected, washed twice with PBS buffer, and resuspended. Genomic DNA was extracted using a nucleic acid extraction kit (Tianjin Xiangxin Medical Device Co., Ltd., Tianjin Medical Device Registration No. 20220543). Briefly, 200 μL of the resuspended solution was incubated with Proteinase K and lysis buffer at 70 °C for 10 minutes. After adding 200 μL of absolute ethanol, the mixture was transferred to a purification column. Following washing steps, DNA was eluted with 53 μL of elution buffer. DNA concentration was quantified using a Qubit 4.0 Fluorometer (Thermo Fisher Scientific, USA). Samples with a DNA concentration ≥ 2.5 ng/μL were used for subsequent detection. Isolated DNA was stored at −20 °C until further analysis.

### 2.4 Mutation detection and result interpretation

The present study actually employed a real-time fluorescent quantitative PCR (qPCR), Mutations were detected using a commercial human FGFR3/TERT mutation detection kit (fluorescent probe-based PCR; Tianjin Xiangxin Medical Device Co., Ltd., China Medical Device Registration No. 20243402136). The kit included specific primers and TaqMan fluorescent probes targeting four hotspot mutations: TERT-M1 (C250T), TERT-M2 (C228T), FGFR3-M1 (p.R248C, c.742C>T), and FGFR3-M2 (p.S249C, c.746C>G), with ACTB (β-actin) serving as the internal reference gene. The detection reaction steps and operations were strictly performed in accordance with the kit instructions.

Interpretation criteria: A sample was considered positive when ΔCt (Ct target – Ct ACTB) was ≤12 for TERT-M1/FGFR3-M1 and ≤11 for TERT-M2/FGFR3-M2, and valid when ACTB Ct ≤28. Two technicians independently performed all tests, with discrepant results resolved by repeat testing.

### 2.5 Statistical analysis

All statistical analyses were performed using SPSS version 26.0 software, and graphs were generated with Python. Continuous variables were expressed as mean ± standard deviation (SD) and compared between groups using the independent-samples t-test. Categorical variables were presented as counts (percentages) and compared using the Pearson chi-square test or Fisher’s exact test. Differences in mutation frequencies across study populations were evaluated using the chi-square test. A p value < 0.05 was considered statistically significant.Pearson’s chi-square test was applied when all expected cell counts in the contingency table were ≥5; when any expected count was <5 or the total number of observations was small (n < 40), Fisher’s exact test was used. P-values in **Table 1** and **Figure 1** were not adjusted for multiple comparisons; given the exploratory, hypothesis-generating nature of the study and the limited subgroup sample sizes, nominal p-values were reported and interpreted cautiously. In **Table 1**, categorical clinicopathological variables were analyzed using Pearson’s chi-square test or Fisher’s exact test according to the rule above, and age was compared using the independent-samples t-test. In **Figure 1**, Fisher’s exact test was used for all categorical comparisons because the early-recurrence group was small (n = 14) and several expected cell counts were <5.

**Table 1:** Associations between mutations and clinicopathological features in 106 newly diagnosed NMIBC patients.

Variables	TERT-M1		TERT-M2		Total TERT-promoter mutations		FGFR3-M1		FGFR3-M2		Total FGFR3 mutations		TERT or FGFR3		TERT and FGFR3	
	No mutations N (%)	≥1 mutations N (%)	No mutations N (%)	≥1 mutations N (%)	No mutations N (%)	≥1 mutations N (%)	No mutations N (%)	≥1 mutations N (%)	No mutations N (%)	≥1 mutations N (%)	No mutations N (%)	≥1 mutations N (%)	No mutations N (%)	≥1 mutations N (%)	No mutations N (%)	≥1 mutations N (%)
All patients	90(84.9%)	16(15.1%)	69(65.1%)	37(34.9%)	54(50.9%)	52(49.1%)	99(93.4%)	7(6.6%)	97(91.5%)	9(8.5%)	90(84.9%)	16(15.1%)	47(44.3%)	59(55.7%)	97(91.5%)	9(8.5%)
Age (years)^a^ Mean ± SD	64.41 ± 11.57	63.25 ± 11.02	63.21 ± 10.87	66.22 ± 12.40	63.20 ± 10.93	65.30 ± 11.97	63.87 ± 11.25	69.43 ± 13.85	64.44 ± 11.77	62.00 ± 7.07	64.06 ± 11.59	65.25 ± 10.86	62.23 ± 11.25	65.83 ± 11.44	64.46 ± 11.33	61.78 ± 13.036
	P=0.895		P=0.368		P=0.344		P=0.820		P=0.184		P=0.836		P=0.682		P=0.681	
sex [n(%)]^b^																
Female	15(75.0%)	5(25.0%)	11(55.0%)	9(45.0%)	7(35.0%)	13(65.0%)	19(95.0%)	1(5.0%)	18(90.0%)	2(10.0%)	17(85.0%)	3(15.0%)	6(30.0%)	14(70.0%)	18(90.0%)	2(10.0%)
Male	75(87.2%)	11(12.8%)	58(67.4%)	28(32.6%)	47(54.7%)	39(45.3%)	80(93.0%)	6(7.0%)	79(91.9%)	7(8.1%)	73(84.9%)	13(15.1%)	41(47.7%)	45(52.3%)	79(91.9%)	7(8.1%)
	P=0.170		P=0.293		P=0.113		P=1.000		P=1.000		P=1.000		P=0.152		P=1.000	
Smoking status^b^																
Smoking history	53(85.5%)	9(14.5%)	41(66.1%)	21(33.9%)	33(53.2%)	29(46.8%	56(90.3%)	6(9.7%)	58(93.5)	4(6.5%)	52(83.9%	10(16.1%)	28(45.2%)	34(54.8%)	57(91.9%)	5(8.1%)
No smoking history	37(84.1%)	7(15.9%)	28(63.6%)	16(36.4%)	21(47.7%)	23(52.3%)	43(97.7%)	1(2.3%)	39(88.6%)	5(11.4%)	38(86.4%)	6(13.6%)	19(43.2%)	25(56.8%)	40(90.9%)	4(9.1%)
	P=0.844		P=0.791		P=0.577		P=0.265		P=0.589		P=0.724		P=0.840		P=0.852	
Number of tumors^b^																
Single	58(89.2%)	7(10.8%)	44(67.7%)	21(32.3%)	37(56.9%)	28(43.1%)	62(95.4%)	3(4.6%)	58(89.2%)	7(10.8%)	55(84.6%)	10(15.4%)	28(45.2%)	34(54.8%)	57(91.9%)	5(8.1%)
Multiple	32(78.0%)	9(22.0%)	25(61.0%)	16(39.0%)	17(41.5%)	24(58.5%)	37(90.2%)	4(9.8%)	39(95.1%)	2(4.9%)	35(85.4%	6(14.6%)	19(43.2%)	25(56.8%)	40(90.9%)	4(9.1%)
	P=0.117		P=0.480		P=0.121		P=0.525		P=0.483		P=0.916		P=0.840		P=1.000	
Tumor diameter^b^																
≤3 cm	75(87.2%)	11(12.8%)	56(65.1%)	30(34.9%)	45(52.3%)	41(47.7%)	81(94.2%)	5(5.8%)	80(93.0%)	6(7.0%)	75(87.2%)	11(12.8%)	31(47.7%)	34(52.3%)	61(93.8%)	4(6.2%)
>3 cm	15(75.0%)	5(25.0%)	13(65.0%)	7(35%)	9(45.0%)	11(55.0%)	18(90.0%)	2(10.0%)	17(85.0%)	3(15%)	15(75.0%)	5(25%)	16(39.0%)	25(61.0%)	36(87.8%)	5(12.2%)
	P=0.170		P=0.992		P=0.555		P=0.858		P=0.475		P=0.170		P=0.382		P=0.466	
T stage^b^																
Ta	48(82.8%)	10(17.2%)	44(75.9%)	14(24.1%)	34(58.6%)	24(41.4%)	55(94.8%)	3(5.2%)	52(89.7%)	6(10.3%)	49(84.5%)	9(15.5%)	39(45.3%)	47(54.7%)	81(94.2%)	5(5.8%)
T1	42(87.5%)	6(12.5%)	25(52.1%)	23(47.9%)	20(41.7%)	28(58.3%)	44(91.7%)	4(8.3%)	45(93.8%)	3(6.3%)	41(85.4%)	7(14.6%)	8(40.0%)	12(60.0%)	16(80.0%)	4(20.0%)
	P=0.479		P=0.011		P=0.082		P=0.795		P=0.507		P=0.894		P=0.664		P=0.109	
Tumor grade^b^																
Low grade	23(85.2%)	4(14.8%)	23(85.2%)	4(14.8%)	19(70.4%)	8(29.6%)	25(92.6%)	2(7.4%)	26(96.3%)	1(3.7%)	24(88.9%)	3(11.1%)	29(50.0%)	29(50.0%)	54(93.1%)	4(6.9%)
High grade	67(84.8%)	12(15.2%)	46(58.2%)	33(41.8%)	35(44.3%)	44(55.7%)	74(93.7%)	5(6.3%)	71(89.9%)	8(10.1%)	66(83.5%)	13(16.5%)	18(37.5%)	30(62.5%)	43(89.6%)	5(10.4%)
	P=1.000		P=0.011		P=0.019		P=1.000		P=0.526		P=0.720		P=0.197		P=0.766	
EAU risk classification(2022)^b^																
Low-and intermediate-risk group	36(80.0%)	9(20.0%)	38(84.4%)	7(15.6%)	29(64.4%)	16(35.6%)	43(95.6%)	2(4.4%)	41(91.1%)	4(8.9%)	39(86.7%)	6(13.3%)	18(66.7%)	9(33.3%)	25(92.6%)	2(7.4%)
High-and very high-risk group	54(88.5%)	7(11.5%)	31(50.8%)	30(49.2%)	25(41.0%)	36(59.0%)	56(91.8%)	5(8.2%)	56(91.8%)	5(8.2%)	51(83.6%)	10(16.4%)	29(36.7%)	50(63.3%)	72(91.1%)	7(8.9%)
	P=0.226		P=0.000		P=0.017		P=0.709		P=1.000		P=0.664		P=0.007		P=1.000	

^a^Continuous variable (age) was described as mean ± standard deviation (SD), and the independent-samples t-test was adopted for inter-group comparison.

^b^Categorical variables including sex, smoking status, tumor number, tumor diameter, T-stage, concomitant CIS, tumour grade and EAU risk classification were presented as n(%). The χ² (chi-square) test was performed for categorical variables when the expected frequency of each cell in the contingency table was ≥ 5.

Fisher’s exact test was applied under the following two conditions: (1) any cell in the contingency table had an expected frequency < 5 while total sample size ≥ 40; (2) the total sample size < 40.

P-value < 0.05 was defined as statistically significant.

**Figure 1:**
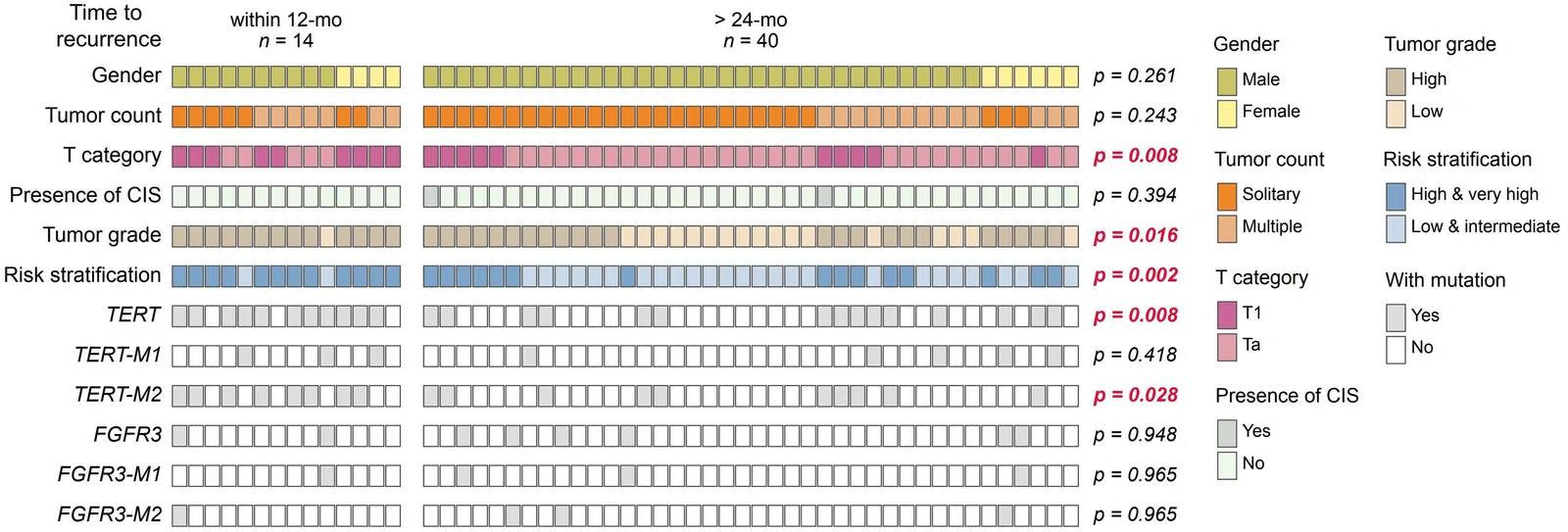
Association of clinicopathological features and TERT/FGFR3 mutations with early recurrence in NMIBC. Patients were stratified into early recurrence (≤12 months, n=14) and long-term recurrence-free group (>24 months, n=40). Each row represents a variable, and each column represents a patient; colored blocks indicate patient-level characteristics. P-values from Fisher’s exact test are shown, with significant associations (p < 0.05) observed for T category, tumor grade, risk stratification, total TERT mutations, and TERT-M2 (C228T).

## 3 Results

### 3.1 Baseline clinicopathological characteristics of the study cohort

A total of 106 patients with newly-diagnosed non-muscle-invasive bladder cancer (NMIBC) from our institution were enrolled in the present study. Baseline clinicopathological features of our internal cohort and three publicly reported external urothelial bladder cancer cohorts are summarized in **Table 2**.

**Table 2:** Baseline clinicopathological characteristics of urothelial bladder cancer patients across four independent cohorts.

Variables	Chinese (n=106)	Italian (n=230)	Japanese (n=59)	French (n=275)
Sex, n (%)				
Female	20 (18.87)	0 (0.00)^a^	11(18.64)	41 (14.91)
Male	86 (81.13)	230 (100.00)	48(81.36)	234 (85.09)
Age, years	64 ± 11 (mean ± SD)	63.8 ± 8.0(mean ± SD)	75(median, 31-91)	74 (median, 34-97)
Smoking, n (%)				
Never-smoker	44 (41.51)	22 (9.57)	NR	NR
Smoker (current/former)	62 (58.49)	208 (90.43)	NR	NR
Tumor number, n (%)				
Single	65 (61.32)	NR	NR	NR
Multiple	41 (38.68)	NR	NR	NR
Tumor diameter, n (%)				
≤3 cm	86 (81.13)	168 (73.04)	NR	NR
>3 cm	20 (18.87)	62 (26.96)	NR	NR
T-stage, n (%)				
Ta	58 (54.72)	147 (63.91)	32(54.24)	199 (72.36)
T1	48 (45.28)	74 (32.17)	27(45.76)	76 (27.64)
≥T2 (MIBC)	—	—	—	—
CIS, n (%)				
Yes	2 (1.89)	7 (3.04)	12(20.34)	12 (4.36)^b^
No	104 (98.11)	223 (96.96)	47(79.66)	263 (95.64)
Rare subtype, n (%)				
Yes	7 (6.60)	NR	NR	NR
No	99 (93.40)	NR	NR	NR
Tumor grade, n (%)				
Low-grade	27 (25.47)	125 (54.35)	20(33.90)	144 (52.36)
High-grade	79 (74.53)	105 (45.65)	39(66.10)	131 (47.64)
Insufficient material	—	—	—	—
EAU risk classification, n (%)				
Low-risk	11 (10.38)	60 (26.09)^c^	NR	NR
Intermediate-risk	34 (32.08)	78 (33.91)^c^	NR	NR
High-risk	49 (46.23)	92 (40.00)^c^	NR	NR
Very high-risk	12 (11.32)	—	NR	NR

Data extracted from original publications; NR = Not reported. Chinese cohort: local cohort of 106 patients with newly-diagnosed NMIBC; Italian cohort (n=230, male-only NMIBC, Critelli et al., Oncotarget, 2016); Japanese cohort (n=114, pre-TURBT samples, includes both NMIBC(n=59) and MIBC(n=55), Hayashi et al., Front Oncol, 2020); French cohort (n=275, NMIBC subset, Descotes et al., Br J Cancer, 2017). Percentages were calculated within each respective cohort.

NR, not reported; SD, standard deviation; MIBC, muscle-invasive bladder cancer; CIS, carcinoma *in situ*; EAU, European Association of Urology.

a Italian cohort exclusively enrolled male patients.

b Twelve patients with concomitant pTis from the overall 348-patient cohort were included in this NMIBC subset.

c Italian cohort used the old-version EAU risk classification without the very-high-risk stratum.

In our cohort, most patients were male (86/106, 81.13%), with a mean age of 64 ± 11 years. Over half of participants had a smoking history (62/106, 58.49%). Most tumors were solitary (65/106, 61.32%) with a maximum diameter ≤3 cm (86/106, 81.13%). Fifty-eight patients (54.72%) presented with Ta-stage disease and 48 (45.28%) with T1-stage disease. Concomitant carcinoma *in situ* (CIS) was noted in 2 patients (1.89%), and rare histological subtypes were seen in 7 patients (6.60%). Twenty-seven patients (25.47%) had low-grade tumors, while 79 (74.53%) had high-grade tumors. Based on the 2022 European Association of Urology (EAU) risk classification, 11 (10.38%), 34 (32.08%), 49 (46.23%), and 12 (11.32%) patients belonged to low-, intermediate-, high-, and very-high-risk subgroups, respectively.

As presented in **Table 2**, the external cohorts differed in patient composition. All participants in the Italian cohort were male NMIBC patients. The French cohort comprised NMIBC cases, whereas the Japanese pre-TURBT cohort contained both NMIBC and muscle-invasive bladder cancer (MIBC) cases. Some clinical parameters (e.g., tumor focality, tumor size, EAU risk category) were not reported in certain published cohorts and are indicated as “NR” in the table.

### 3.2 Mutation frequencies of TERT promoter and FGFR3 hotspots in urinary DNA

Mutation analysis of urinary exfoliated cell DNA from 106 patients with NMIBC is presented in **Figure 2**. The overall mutation rate of the TERT promoter was 49.06% (52/106), including TERT-M1 (C250T) in 15.09% (16/106) and TERT-M2(C228T) in 34.91% (37/106). The overall FGFR3 mutation rate was 15.09% (16/106), comprising FGFR3-M1 (p.R248C) in 6.60% (7/106) and FGFR3-M2 (p.S249C) in 8.49% (9/106). The positivity rate for any mutation was 55.66% (59/106), and the rate of concurrent TERT and FGFR3 mutations was 8.49% (9/106).

**Figure 2:**
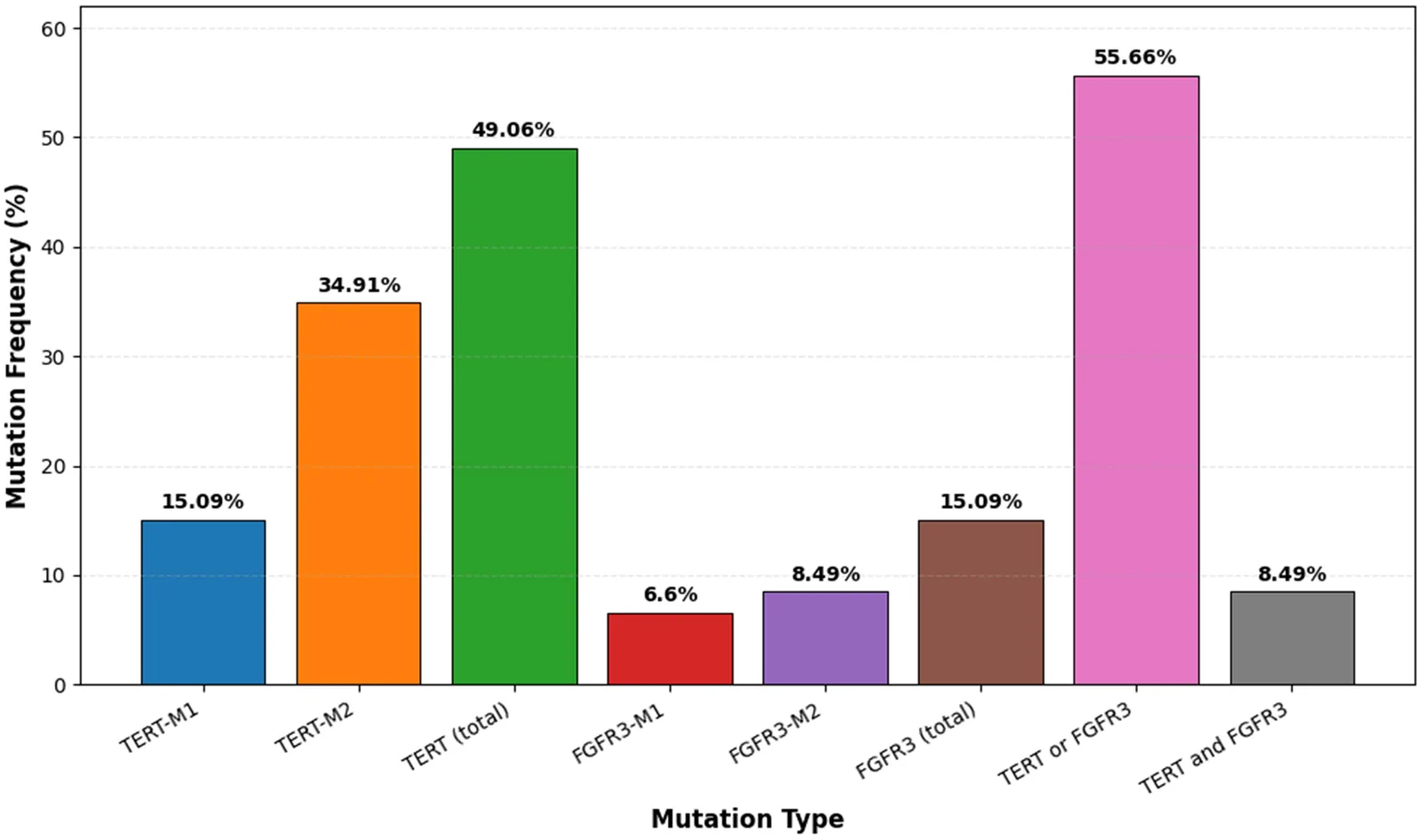
Mutation frequencies of TERT promoter and FGFR3 hotspot mutations in urine-derived DNA from 106 bladder cancer patients. Mutations include individual (TERT-M1/C250T, TERT-M2/C228T, FGFR3-M1/p.R248C and FGFR3-M2/p.S249C) and combined (total TERT, total FGFR3, TERT or FGFR3, and concurrent TERT and FGFR3) variants. The y-axis shows mutation rate, with exact values labeled above bars.

### 3.3 Comparative analysis of NMIBC mutation frequencies across diverse populations

The results of the present study were compared with those from published NMIBC cohorts in Japan, France, and Italy, as illustrated in **Figure 3**. The Japanese cohort (Hayashi et al., 2020) included 59 NMIBC patients, in whom urinary cell-free DNA (cfDNA) was analyzed using droplet digital PCR (ddPCR). The detected mutation rates were 13.60% for TERT-M1, 47.50% for TERT-M2, and 32.30% for total FGFR3; the total TERT mutation frequency was unavailable in this study (11). The French cohort (Descotes et al., 2017) enrolled 275 NMIBC patients evaluated by Sanger sequencing of urinary exfoliative cells. The TERT-M1 mutation rate was 10.10%, the TERT-M2 mutation rate reached 66.55%, and the overall TERT mutation rate was 79.44%; no FGFR3 subtype or total FGFR3 mutation data were reported (12). Of the 230 male NMIBC patients in the Italian cohort (Critelli R et al., 2016), 113 had available urinary exfoliative-cell DNA for Sanger-based mutation analysis. In this molecular subset, the TERT-M1 rate was 8.26%, TERT-M2 rate was 50.87%, overall TERT mutation rate was 51.74%, FGFR3-M1 rate was 4.35%, FGFR3-M2 rate was 22.17%, and overall FGFR3 mutation rate was 41.30% (13).

**Figure 3:**
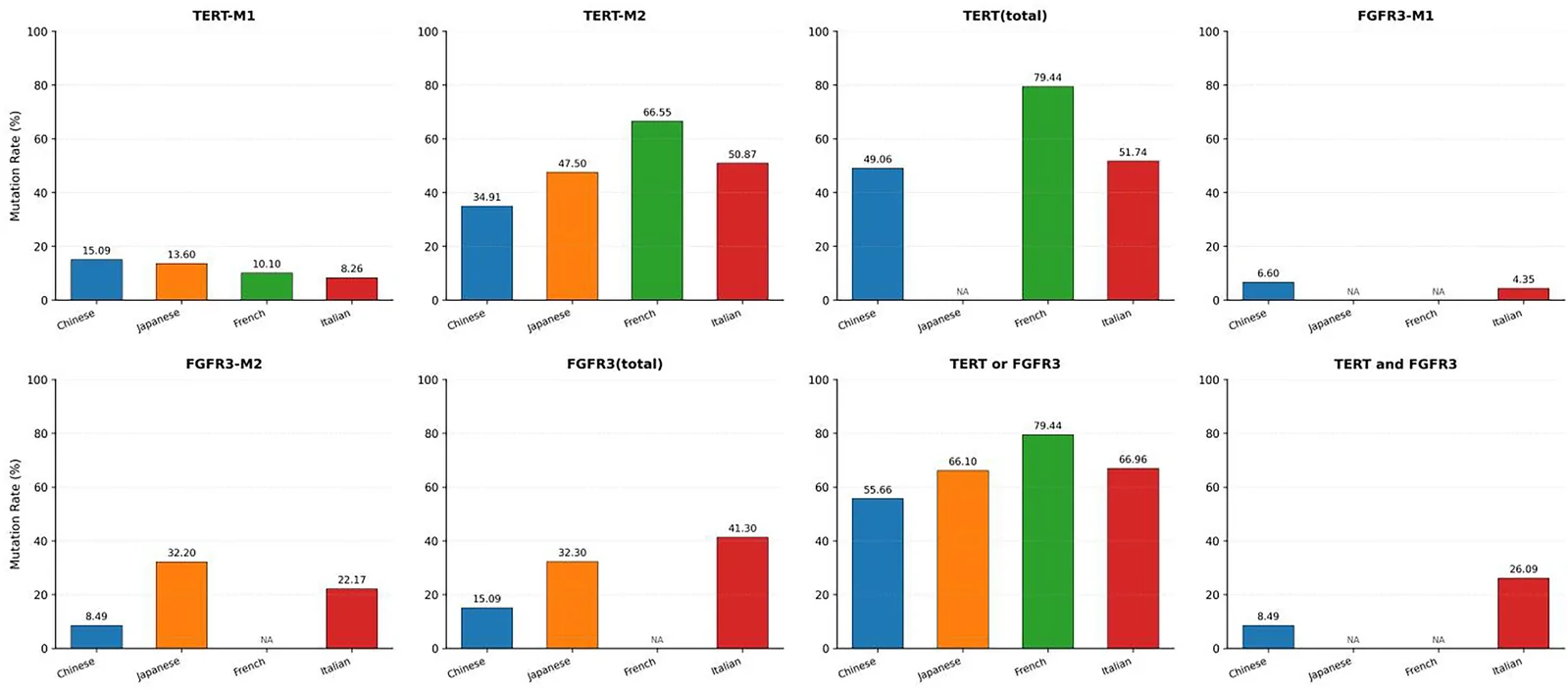
Comparison of TERT and FGFR3 mutation rates among NMIBC patients across four study cohorts: Chinese (current study, n=106), Japanese (Hayashi et al., 2020), French (Descotes et al., 2017), and Italian (Critelli R et al., 2016). Each subplot represents a distinct mutation category. The y-axis shows the mutation rate. “NA” indicates unavailable data in published studies. In the Japanese cohort, FGFR3-M2 was not assayed; the reported FGFR3 "total" frequency therefore equals the FGFR3-M1 frequency only and should not be interpreted as a true combined FGFR3 mutation rate.

Comparative analysis of mutation frequencies revealed population-specific differences in the urinary tumor DNA (utDNA) molecular profile, with the Chinese cohort consistently displaying lower frequencies of the key hotspot mutations. Specifically, the TERT-M2 (C228T) mutation rate in our Chinese cohort (34.91%) was lower than those reported in the French (66.55%), Italian (50.87%), and Japanese (47.50%) study cohorts. The overall FGFR3 mutation rate (15.09%) was also lower than those reported in the Italian (41.30%) and Japanese (32.30%) study cohorts. By contrast, the overall TERT promoter mutation rate in our Chinese cohort (49.06%) was similar to that of the Italian cohort (51.74%) but lower than that of the French cohort (79.44%). Total TERT mutation frequency data were not reported for the Japanese cohort, which precluded cross-cohort comparison. Given the methodological and clinicopathological heterogeneity among the studies, these comparisons were descriptive, and direct statistical testing was not performed.

### 3.4 Correlations between TERT/FGFR3 mutations and clinicopathological characteristics

Associations between individual mutation loci and clinicopathological features were analyzed (**Table 1**). The TERT-M2 mutation was significantly associated with T stage, tumor grade, and EAU risk stratification (all P < 0.05). Specifically, the mutation rate was higher in T1 versus Ta tumors (47.9% vs. 24.1%, P = 0.011), high-grade versus low-grade tumors (41.8% vs. 14.8%, P = 0.011), and high/very high-risk versus low/intermediate-risk groups (49.2% vs. 15.6%, P < 0.001). Overall, TERT promoter mutations were significantly associated with tumor grade (55.7% vs. 29.6%, P = 0.019) and EAU risk stratification (59.0% vs. 35.6%, P = 0.017), and showed a non-significant positive trend with T stage (P = 0.082). Combined TERT/FGFR3 mutations were also significantly associated with tumor grade and EAU risk stratification (both P < 0.01). The combined mutation rate was higher in high-grade tumors (63.3% vs. 33.3%, P = 0.007) and patients in the high/very high-risk category (67.2% vs. 40.0%, P = 0.005). No significant association was identified for T stage (P = 0.197).

No significant associations were observed between TERT-M1, FGFR3-M1, FGFR3-M2, total FGFR3 mutations, or TERT/FGFR3 co-mutations and age, sex, smoking history, tumor number, tumor size, T stage, tumor grade, or EAU risk stratification (all P > 0.05).

### 3.5 Potential association with recurrence

A total of 106 patients with NMIBC were enrolled. Among them, 54 patients with complete follow-up data met the predefined stratification criteria and were included in the comparative analysis. Fourteen patients experienced recurrence or progression within 12 months after TURBT (early recurrence group), whereas 40 patients remained recurrence- and progression-free for more than 24 months (long-term recurrence-free group). Comparison of clinicopathological and molecular features between the two groups (**Figure 1**) revealed no significant differences in sex, tumor multiplicity, presence of carcinoma *in situ* (CIS), or FGFR3 mutations and their subtypes (all P > 0.05). In contrast, T1 stage, high-grade tumors, and high/very-high risk stratification were significantly more prevalent in the early recurrence group than in the long-term recurrence-free group (P = 0.008, 0.016, and 0.002, respectively). At the molecular level, the overall TERT promoter mutation rate and the TERT-M2 subtype mutation rate were significantly higher in the early recurrence group than in the long-term recurrence-free group (P = 0.008 and 0.028, respectively), whereas no significant difference was observed in the TERT-M1 subtype between the two groups. These findings indicate that, under stringent stratification, adverse clinicopathological features and TERT promoter mutations-particularly the TERT-M2 subtype-are enriched in patients with early recurrence. Within this cohort, TERT promoter mutations, especially TERT-M2, were associated with early NMIBC recurrence. This observation may complement conventional clinical risk factors and help inform recurrence risk assessment and postoperative management decisions, although validation in larger independent cohorts is required.This finding aligns with the study by Tsukahara et al., which also demonstrated the predictive value of urinary TERT promoter mutations for NMIBC recurrence (14).

## 4 Discussion

Bladder cancer exhibits significant genomic heterogeneity across different ethnicities, which poses a critical barrier to the clinical translation and application of molecular markers and contributes to the inconsistent therapeutic outcomes observed among diverse populations (6). Using a prospective cohort, this study systematically characterizes TERT promoter and FGFR3 hotspot mutations detected in urinary tumor DNA (utDNA) from Chinese patients with newly diagnosed NMIBC. We observed a mutational pattern in which the TERT-M2 mutation was associated with aggressive clinicopathological features.When these data are viewed alongside those of previously published cohorts, however, several caveats should be borne in mind. Given the substantial differences in detection platforms, urine collection and processing, DNA input, and patient characteristics across studies, mutation rates derived from different cohorts are not directly comparable. We therefore did not perform statistical comparisons between the Chinese cohort and the French, Italian, or Japanese cohorts, and the previously published mutation frequencies are presented alongside ours solely for reference (**Figure 3**). The observed inter-cohort differences in mutation frequency should thus be interpreted with caution, as they may reflect the combined effects of biological factors (i.e., study population profiles and clinicopathological characteristics) and technical and preanalytical heterogeneity.Collectively, these observations add supporting evidence for non-invasive molecular testing and recurrence risk assessment among Chinese patients with NMIBC.Future multicenter studies employing standardized detection platforms and harmonized preanalytical protocols are warranted to validate these findings and to further explore population-related factors shaping the utDNA mutation profile.

### 4.1 Population-related features of TERT promoter and FGFR3 mutations in Chinese patients with NMIBC

TERT promoter and FGFR3 are the most prevalent core driver genes in bladder cancer, with their mutation frequencies and subtype distributions varying significantly across ethnic groups (15). To investigate this, we compared the mutation profiles of our Chinese cohort with those reported in NMIBC cohorts from Italy (Caucasian) (13), France (Caucasian) (12), and Japan (East Asian) (11). We found that the overall mutation rates of the TERT promoter (49.06%) and FGFR3 (15.09%) in Chinese patients were significantly lower than those observed in the Japanese, Italian, and French populations, consistent with previous molecular studies in Asian cohorts (15).

Of note, we observed differences in the distribution of TERT mutation subtypes among the included cohorts. In Chinese patients, TERT-M2 (C228T) was the predominant subtype, accounting for 71.2% of all TERT mutations, whereas TERT-M1 (C250T) predominated in the French, Italian, and Japanese cohorts included for comparison. Notably, differences in mutation profiles were also observed between the Chinese and Japanese cohorts, both of which comprise East Asian populations. These inter-cohort variations should be interpreted with caution, given potential differences in patient selection, detection platforms, and preanalytical procedures across studies.

This discrepancy may arise from two primary factors. First, the unique genomic background of the Chinese population: genetic polymorphisms at rs2853669 and rs2853670 in the TERT promoter region may increase susceptibility to the TERT-M2 (C228T) mutation (16). Second, environmental exposures, such as smoking patterns and occupational hazards (e.g., aromatic amines and heavy metals), differ considerably between Chinese and Caucasian populations, thereby shaping distinct mutational signatures (17).

Furthermore, recent studies have demonstrated that activating TERT promoter mutations are significantly associated with smoking and age, suggesting that tobacco exposure directly promotes the clonal expansion of TERT-mutant cells in normal urothelium. Additionally, truncating mutations in multiple driver genes occur significantly more frequently in male bladder epithelium than in females, further highlighting the regulatory role of sex-related genetic background in mutational selection (18). Nevertheless, the biological and functional differences between distinct TERT mutation subtypes remain to be fully elucidated.

Notably, inter-cohort differences in clinicopathological features represent a major driver of divergent molecular alteration profiles across NMIBC study populations. Our cohort was recruited from a tertiary referral cancer center, in which 74.53% of enrolled patients presented with high-grade NMIBC. Given that FGFR3 alterations are well-established molecular hallmarks of low-grade NMIBC, the high proportion of high-grade tumors provides a plausible pathological explanation for the low FGFR3 mutational frequency observed in our work. Differences in case composition also partially account for the moderate discrepancy in the overall TERT mutation rate (49.06%) compared with Western cohorts dominated by screen-detected low-grade NMIBC. In addition to clinicopathological confounding factors, genetic heterogeneity across study cohorts may contribute to variations in mutation profiles. In the present study, the overall FGFR3 mutation rate among Chinese patients with NMIBC was 15.09%. This value was lower than those reported in Japanese (32.3%) and Italian (41.3%) study cohorts, with low prevalence observed for both FGFR3-M1 and FGFR3-M2 variants. FGFR3 mutations are known to preferentially occur in low-grade, low-stage NMIBC (19), and genetic heterogeneity across study populations may contribute to differences in FGFR3 mutation hotspots.Indeed, previous studies have revealed fundamental disparities in FGFR3 mutational spectra and its downstream signaling pathways between Chinese and Western populations (6), implying that FGFR3 may not serve as a core driver gene for NMIBC in Chinese patients. Accordingly, FGFR3-targeted therapies developed for Caucasian individuals cannot be directly extrapolated to Chinese populations, highlighting the clinical demand for targeted agents optimized for Chinese-specific mutational features.

Our cohort yielded a TERT/FGFR3 combined positive rate of 55.66%, which is lower than the rates reported in Japanese (66.1%) and Italian (69.1%) NMIBC populations, largely due to the intrinsically low FGFR3 mutational prevalence in Chinese patients. Integrating additional bladder cancer driver genes (e.g., TP53, KMT2D) into current urinary panels is therefore necessary to enhance detection sensitivity, offering a promising strategy for future liquid biopsy optimization (20). Although this two-gene utDNA panel achieved reliable diagnostic performance in our prospective Chinese cohort, further improvements are required for large-scale clinical implementation. The current detection platform is a cost-effective and clinically accessible approach for routine molecular screening. However, conventional relative quantitative PCR has inherent limitations in detecting ultra-low-abundance urinary tumor DNA, restricting the diagnostic capacity of the dual-gene panel. In addition to genomic expansion, technical innovation is indispensable for performance enhancement. Future translational studies can focus on developing a multi-channel fluorescence-enabled multiplex ddPCR panel tailored for the Chinese population. Leveraging ddPCR’s unique advantages in single-molecule absolute quantification and anti-interference capability, this optimized multi-gene detection system can substantially improve diagnostic sensitivity and stability, thereby promoting the standardized and widespread application of non-invasive utDNA screening in primary clinical settings.

### 4.2 Correlations between TERT promoter and FGFR3 mutations and clinicopathological features

Association analyses revealed that TERT-M2 (C228T) was the only mutation significantly associated with multiple clinicopathological parameters. TERT-M2 was associated with T1 stage, high-grade histology, and high/very high-risk EAU stratification (all P < 0.05), indicating an association with aggressive tumor features among Chinese patients with NMIBC. In contrast, TERT-M1 exhibited no significant association with any clinicopathological variable.

Notably, these results differ from Western studies, in which both TERT-M1 and TERT-M2 mutations are common but show no specific association with tumor stage or grade (21). Therefore, TERT-M2 may be associated with tumor progression in Chinese patients with NMIBC, and may provide a candidate molecular marker for further investigation in risk assessment and disease monitoring.

Mechanistically, TERT promoter mutations sustain unlimited proliferation and clonal expansion through telomerase activation. Importantly, accumulating evidence suggests that the TERT-M2 mutation may activate telomerase more efficiently than TERT-M1 (22), which could explain its association with more aggressive tumor behavior.

Additionally, total TERT mutations and combined TERT/FGFR3 mutations were significantly associated with high-grade tumors and high/very high-risk EAU stratification (all P < 0.05), consistent with previous reports. Specifically, Descotes et al. found that TERT mutations increased the recurrence risk of NMIBC by 5.34-fold, even in patients with negative cystoscopy (12). Similarly, Batista et al. linked TERT promoter mutations to an elevated recurrence risk following BCG therapy (23). Therefore, these mutations may act as candidate molecular markers to help assess tumor malignancy among Chinese patients with NMIBC and may improve risk stratification when integrated with conventional clinicopathological features.

In our cohort, FGFR3 mutations showed no significant correlation with any clinicopathological parameter. Although FGFR3 mutation rates were slightly higher in low-grade, Ta, and low/intermediate-risk groups, the differences were not statistically significant—in sharp contrast to the strong association between FGFR3 mutations and low-grade/Ta NMIBC in Caucasians (24). Collectively, these observations suggest heterogeneity in the clinical relevance of FGFR3 across study populations, which is likely related to its globally low mutation rate in Chinese patients (25). Ultimately, its clinical value in Chinese NMIBC requires validation in larger cohorts.

### 4.3 Potential association of TERT promoter and FGFR3 mutations with recurrence

The present study explored the association between urinary TERT promoter and FGFR3 hotspot mutations and recurrence in Chinese patients with newly diagnosed NMIBC, using an extreme phenotypic stratification strategy. Different from conventional cohort analyses with continuous follow-up outcomes, the current design strictly enrolled two representative subgroups: patients with early recurrence/progression within 12 months and patients maintaining long-term recurrence-free status for more than 24 months. This extreme grouping approach enhanced molecular and clinical phenotypic differences and improved statistical sensitivity for identifying candidate molecular markers associated with recurrence, although it excluded intermediate cases with recurrence occurring between 12 and 24 months. Therefore, the associations between TERT and FGFR3 mutations and recurrence identified in this stratified cohort require further verification in large-sample, real-world, full-spectrum NMIBC populations to determine their broader clinical utility.

Accumulated studies have validated that TERT promoter mutation serves as a core molecular biomarker for evaluating NMIBC recurrence risk with reliable prognostic performance (14). Consistent with previous findings, the current study observed enrichment of overall TERT promoter mutations and the TERT-M2 subtype among patients with early recurrence, suggesting associations with aggressive tumor phenotypes and adverse clinical outcomes. Notably, the TERT-M1 subtype did not show a significant association with recurrence, indicating functional heterogeneity among different TERT mutation subtypes. Combined with our cross-population comparison results, the mutational profiles of TERT and FGFR3 show heterogeneity across Chinese, Japanese, French, and Italian NMIBC study cohorts. Although our Chinese cohort exhibited a lower TERT-M2 mutation rate (34.91%) compared with Japanese (47.50%), French (66.55%), and Italian (50.87%) study populations, TERT-M2 remained the predominant TERT mutation subtype in Chinese patients and was correlated with high-risk clinicopathological features. This molecular pattern differs from the high TERT mutation prevalence reported in Caucasian study cohorts, reflecting differing molecular pathogenetic features in Chinese patients with NMIBC. Collectively, these observations point to an association between the TERT-M2 subtype and recurrence within our Chinese cohort; further large-scale studies are required to validate its potential utility for postoperative risk stratification in this patient group.

From the perspective of detection methodology, this study adopted PCR-based urinary liquid biopsy for hotspot mutation detection. Our qPCR assay differs from the Sanger sequencing utilized in the French cohort (Descotes et al., 2017) and Italian cohort (Critelli et al., 2016), as well as the ddPCR platform applied in the Japanese cohort (Hayashi et al., 2020). Nevertheless, the analytical and clinical performance of this qPCR platform has been validated in prior work. During kit development, concordance against Sanger sequencing was evaluated in a double-blinded, multicenter prospective trial assessing multi-target urinary DNA assays for urothelial carcinoma, which confirmed favorable diagnostic sensitivity and specificity of the assay (26). As a robust molecular detection technique, qPCR possesses prominent technical strengths including simple operation, low cost, high detection sensitivity, stable quantitative results, and favorable repeatability, without reliance on complex bioinformatic analysis (27). These characteristics make qPCR well-suited for widespread clinical screening and long-term postoperative surveillance, particularly in primary-care settings. As a non-invasive and repeatedly applicable test, urinary mutation detection can serve as a complementary tool to cystoscopy, alleviating patient discomfort and suboptimal compliance associated with invasive endoscopic procedures, and facilitating improved overall diagnostic and prognostic performance in real-world clinical practice.

Several unavoidable limitations of the present study should be addressed. First, the lack of matched TURBT tumor tissue precluded direct assessment of mutation concordance and of utDNA sensitivity against tissue gold-standard genomics. Nevertheless, the biological validity of our urinary findings is supported by earlier work showing that FGFR3, TERT promoter, TP53, and ERBB2 mutations in urine were also present in matched tumor tissue, although not all tissue mutations were captured in urine (Yang et al., 2023) (28), data that also guided the current two-gene panel. Moreover, our utDNA-derived mutation frequencies were consistent with published NMIBC tissue data. Together, these findings suggest the potential utility of utDNA as a non-invasive approach; however, paired urine-tissue validation in larger cohorts is required before its clinical application can be established, particularly in settings where tissue sampling is unavailable.Second, the single-center nature and limited sample size restrict the generalizability of the conclusions, and multi-center prospective validation is required in subsequent studies. Third, the follow-up duration was relatively short, failing to evaluate the long-term prognostic outcomes of enrolled patients, including long-term recurrence, tumor progression, and overall survival. Fourth, this study did not explore the potential association between TERT/FGFR3 mutational status and the efficacy of intravesical chemotherapy or immunotherapy; thus, the guiding value of these molecular markers for individualized treatment remains unclear. Fifth, the consistency of mutation detection between urinary DNA and plasma cell-free DNA was not compared, and multi-specimen joint detection strategies can be optimized in future studies to improve biomarker detection efficiency. Sixth, although ARMS-qPCR achieves broadly comparable analytical performance in detecting TERT and FGFR3 hotspot mutations relative to Sanger sequencing (French and Italian cohorts) and ddPCR (Japanese cohort), inter-cohort heterogeneity in preanalytical parameters, including urine collection, sample processing, and DNA input, may still introduce potential confounding bias. Mutation frequencies from our cohort and those extracted from previously published external cohorts are presented purely for descriptive purposes and readers’ reference only. No formal statistical analyses or inter-cohort comparative tests were performed, and no statistical conclusions were drawn from these cross-study-population datasets.

In conclusion, urinary TERT and FGFR3 hotspot mutations among Chinese patients with NMIBC show inter-cohort differences compared with published datasets. Although the TERT-M2 mutation rate in our cohort is lower than those reported in Western and Japanese study cohorts, the TERT-M2 subtype is associated with high-risk clinicopathological phenotypes and early tumor recurrence in this patient group. The PCR-based non-invasive urinary mutation assay may serve as a feasible and efficient candidate tool for molecular risk assessment and postoperative surveillance of NMIBC in Chinese patients. Large-scale, multicenter, long-term prospective cohorts are required to validate the consistency of these mutation associations and their clinical utility, and to further explore their potential role in supporting individualized treatment decision-making.

## Data Availability

The raw data supporting the conclusions of this article will be made available by the authors, without undue reservation.
